# Obstructive Sleep Apnea and Pulmonary Embolism: Prevalence, Severity and the Association of Sleep-Disordered Breathing with Thromboembolic Disease

**DOI:** 10.3390/jcm15207892

**Published:** 2026-10-13

**Authors:** Patricia Pérez-Ferrer, Eusebi Chiner, Ignacio Boira, Juan José Piqueras, Adela Martínez-Sanchis, Luis Hernández-Blasco

**Affiliations:** 1 Pneumology Department, Marina Baixa University Hospital, 03570 Alicante, Spain; perezferrerpatricia@gmail.com (P.P.-F.); martinez_adesan@gva.es (A.M.-S.); 2 Pneumology Department, San Juan de Alicante University Hospital, 03550 Alicante, Spain; echinervives@gmail.com; 3 Department of Clinical Medicine, Miguel Hernández University, 03550 Alicante, Spain; lhernandez@umh.es; 4 Molecular Biology Department, Instituto Bernabeu, 03016 Alicante, Spain; jjpiqueras@institutobernabeu.com; 5 Pneumology Department, Dr. Balmis General University Hospital, 03010 Alicante, Spain

**Keywords:** obstructive sleep apnea, pulmonary embolism, venous thromboembolism, apnea–hypopnea index, sleep-disordered breathing, deep vein thrombosis, anticoagulation, thromboembolic recurrence, intermittent hypoxia, respiratory polygraphy

## Abstract

**Background/Objectives:** Obstructive sleep apnea (OSA) and pulmonary embolism (PE) share several risk factors, while emerging evidence suggests a potential pathophysiological link beyond these common determinants. We aimed to characterize the prevalence and severity of OSA in patients with acute PE and to investigate whether the apnea–hypopnea index (AHI) is independently associated with PE and its clinical course. **Methods:** In this prospective, single-center, case–control study, 128 consecutive patients with acute PE and 252 outpatient controls with other respiratory diseases underwent home respiratory polygraphy. In patients with PE, the sleep study was performed at least three months after the acute event. OSA was diagnosed and graded according to international consensus criteria. Logistic regression models were used to evaluate associations between AHI and PE, acute PE severity, chronic deep vein thrombosis (DVT), and venous thromboembolism (VTE) recurrence. **Results:** OSA was present in 55.5% of patients with PE, with severe OSA in 28.9%, compared with 42.1% and 13.9%, respectively, among controls (*p* = 0.013 and *p* = 0.001). Patients with PE had a higher AHI (21.21 ± 19.29 vs. 14.81 ± 14.26 events/h; *p* = 0.007) and oxygen desaturation index (19.86 ± 18.77 vs. 13.30 ± 13.79; *p* = 0.003). Each 10-events/h increase in AHI was independently associated with PE after adjustment for age, sex, BMI, and established VTE risk factors (OR 1.205, 95% CI 1.019–1.426; *p* = 0.029). AHI was not independently associated with intermediate–high/high-risk PE or chronic DVT. Among patients who discontinued anticoagulation, VTE recurrence was higher in those with OSA (36.0% vs. 13.3%; *p* = 0.049). **Conclusions:** OSA is highly prevalent and frequently severe in patients with acute PE. Increasing AHI is independently associated with the presence of PE, supporting a clinically relevant association between sleep-disordered breathing and thromboembolic disease. Because OSA was not systematically reassessed after documented resolution of PE, persistence of OSA independently of residual thromboembolic disease cannot be definitively established. Whether the observed association between OSA and recurrent VTE after anticoagulation withdrawal is confirmed in larger prospective studies remains to be determined.

## 1. Introduction

Pulmonary thromboembolism (PE) is an important cause of morbidity and mortality worldwide, with an estimated annual incidence between 39 and 115 per 100,000 inhabitants, and represents the third-most-frequent cause of cardiovascular death [1,2]. Although numerous risk factors are well known, more than one third of episodes occur in the absence of identifiable triggering factors and are classified as unprovoked PE, a circumstance associated with a higher risk of recurrence after the discontinuation of anticoagulation [3].

Obstructive sleep apnea (OSA) is a highly prevalent and underdiagnosed disease, with prevalence estimates between 9% and 38% in the general population [4]. It is characterized by recurrent episodes of upper-airway collapse during sleep that produce intermittent hypoxia, sleep fragmentation, and changes in intrathoracic pressure. These phenomena favor mechanisms such as systemic inflammation, endothelial dysfunction, and hypercoagulability, providing a plausible pathophysiological basis for a possible association between OSA and venous thromboembolic disease (VTE) [5,6,7].

The available evidence on this relationship remains limited. Although both diseases share risk factors, such as advanced age and obesity, there is growing evidence that OSA may be independently associated with VTE. Several studies have described a higher prevalence of OSA in patients with acute PE and have suggested a possible association with the greater severity and recurrence of VTE, although the results are heterogeneous and are conditioned by multiple confounding factors [6,7,8]. In addition, recent studies suggest that nocturnal hypoxemia indicators may be more closely associated with thromboembolic risk than the apnea–hypopnea index (AHI), whose independent association with PE remains controversial [9,10].

Given the limited number of prospective studies, the main objective was to estimate the prevalence and severity of OSA in patients with acute PE, and to analyze the independent association between the AHI and the presence of PE. As secondary objectives, the association of the AHI with the initial severity of PE, thromboembolic chronicity, and VTE recurrence during follow-up was evaluated, and the prevalence and severity of OSA was compared with those observed in a control group of outpatient patients with other respiratory diseases.

The present study aimed not only to assess the prevalence and severity of OSA in patients with PE, but also to compare these findings with a respiratory control group undergoing the same standardized respiratory polygraphy protocol and to determine whether AHI was independently associated with PE after adjustment for relevant confounding factors.

## 2. Materials and Methods

### 2.1. Study Design

A prospective, single-center, case–control study was carried out in the Pulmonology Department of Hospital Marina Baixa (Spain).

The cases included were patients older than 18 years admitted consecutively to the Pulmonology Department with a diagnosis of acute PE confirmed by computed tomography pulmonary angiography (CT-PA) from July 2023 to July 2025. Exclusion criteria were age > 80 years, prior diagnosis of OSA, refusal to participate, inability to understand the study due to psychiatric pathology or cognitive impairment, impossibility of outpatient follow-up, or estimated survival of less than 12 months.

The control group consisted of patients older than 18 years followed in outpatient pulmonology clinics for respiratory diseases other than PE. This group was selected to provide a clinically relevant respiratory comparison population rather than a general-population control group. Controls were recruited during the same study period as the patients with PE, and no individual matching was performed. Exclusion criteria were age > 80 years, prior history of VTE or OSA, presence of advanced cardiorespiratory disease with chronic respiratory failure, refusal to participate, inability to understand the study due to psychiatric pathology or cognitive impairment, impossibility of outpatient follow-up, or estimated survival less than 12 months.

The study protocol was approved by the Clinical Research Ethics Committee of University Hospital San Juan de Alicante at its meeting on 27 June 2023 (approval code: 23/045), and all participants gave written informed consent.

### 2.2. Variables and Measurements

In the case group, during hospitalization, selection criteria were verified and informed consent was obtained. At this first visit, a protocolized questionnaire was completed that included clinical history, toxic habits, comorbidities, usual medication, demographic and anthropometric variables (age, sex, and BMI), risk factors for PE, and symptoms related to OSA. Daytime sleepiness was evaluated using the Epworth Sleepiness Scale (ESS), considering pathological a score > 10 [11]. PE presenting symptoms, vital signs on admission (blood pressure, heart rate, and oxygen saturation (SpO_2_)), cardiac biomarkers (NT-proBNP and high-sensitivity troponin T), CT-PA findings, and transthoracic echocardiography (TTE) findings were recorded, where the presence of right ventricular (RV) dysfunction was evaluated, as was the presence or absence of concomitant deep vein thrombosis (DVT) on lower limb ultrasound. Clinical severity was evaluated using the Pulmonary Embolism Severity Index (PESI) [12], considering classes III-V as higher-risk categories. Prognostic stratification was performed according to the European Society of Cardiology (ESC) classification, differentiating low, intermediate–low, intermediate–high, and high risk [13]. During admission, anticoagulant treatment was administered, with thrombolysis indicated in selected cases of high-risk PE with hemodynamic instability. Oral anticoagulation was maintained for a minimum of 3–6 months following the recommendations of the international PE guidelines [13]. A follow-up visit was performed at 3–6 months from the start of anticoagulation. At that visit, a CT-PA was performed in all patients to evaluate resolution or chronicity of PE. In those with DVT, a lower limb ultrasound was performed to assess resolution or persistence, and in patients with prior RV dysfunction, a control echocardiogram. Likewise, complete pulmonary function tests, a thrombophilia study (antiphospholipid antibodies, protein C and protein S levels, antithrombin levels, and testing for factor V Leiden and prothrombin gene mutation, when clinically applicable), and home respiratory polygraphy were performed. At this visit, it was decided whether to discontinue or maintain anticoagulation indefinitely. In those patients with persistent PE, DVT, or RV dysfunction, the respective imaging tests were repeated at one year of follow-up to determine persistence or resolution of the abnormalities. On the other hand, diagnosis and severity classification of OSA were performed according to the International Consensus Document on Obstructive Sleep Apnea [14], and treatment with continuous positive airway pressure (CPAP) was indicated according to the recommendations of the same consensus. Subsequently, cases were followed for 12 months and all-cause mortality and thrombotic recurrence were recorded.

In the control group, during the first visit, selection criteria were verified and informed consent was obtained. Toxic habits, comorbidities, usual medication, demographic and anthropometric characteristics, and PE risk factors were recorded. All included controls underwent home respiratory polygraphy, and, after it was performed, the OSA symptom collection protocol, including the ESS, was completed. Diagnosis, severity classification, and indication for CPAP treatment were established according to the same document [14].

#### 2.2.1. Sleep Study

All included patients, cases and controls, underwent a sleep study. Respiratory polygraphy was performed during the 3–6-month follow-up visit, concurrently with CT-PA assessment of PE resolution, in order to minimize the possible influence of the acute phase of PE on the frequency of obstructive respiratory events during sleep, particularly described in patients with right ventricular dysfunction [15,16]. It was not systematically repeated after a documented resolution of PE.

In cases, the study was performed at least three months after hospitalization, in order to minimize the possible influence of the acute phase of PE on the frequency of obstructive respiratory events during sleep, particularly described in patients with right ventricular dysfunction [15,16].

The sleep study was performed at home with a respiratory polygraphy validated against polysomnography (PSG). Type III portable devices (Alice PDx, Philips Respironics, Murrysville, PA, USA) were used, which recorded nasal flow and snoring using a nasal cannula, respiratory effort using thoracoabdominal plethysmographic bands, oxygen saturation and heart rate using pulse oximetry, and body position. The recordings were manually analyzed by hospital pulmonologists who were unaware of the clinical characteristics of the patients and were considered valid when the study duration was greater than 4 h.

Respiratory events during sleep were defined according to current guidelines [17]. The apnea–hypopnea index (AHI) was defined as the total number of apneas and hypopneas per hour of recording time. The oxygen desaturation index (ODI) was defined as the number of ≥3% oxygen desaturations per hour of recording, and TC90 as the percentage of recording time with SpO_2_ < 90%. According to the International Consensus Document on Obstructive Sleep Apnea [14], the diagnosis of OSA was established when any of the following criteria were met: (1) presence of an AHI ≥ 15 events/h, with predominance of obstructive events; (2) presence of an AHI ≥ 5 events/h accompanied by compatible symptoms: excessive daytime sleepiness, non-restorative sleep, excessive tiredness and/or impaired sleep-related quality of life, not justifiable by other causes. OSA severity was classified as mild (AHI 5–14.9), moderate (AHI 15–29.9), or severe (AHI ≥ 30 events/h).

CPAP was prescribed according to the International Consensus Document [14] when indicated. CPAP adherence during follow-up was not systematically assessed and was therefore not included in the statistical analyses.

#### 2.2.2. Transthoracic Echocardiography (TTE)

All patients in the case group underwent a TTE, usually during the first 48 h after the diagnosis of PE. RV dysfunction was considered when at least one of the following echocardiographic findings was identified: RV dilation (RV end-diastolic diameter > 30 mm in the parasternal view or RV/LV diameter ratio ≥ 1 in the apical four-chamber view); hypokinesia of the RV free wall in any of the evaluated projections; or an estimated systolic pulmonary artery pressure (SPAP) ≥ 40 mmHg. Other echocardiographic parameters evaluated were tricuspid annular plane systolic excursion (TAPSE), interventricular septum flattening, inferior vena cava diameter, presence of intracavitary thrombi, and maximum systolic velocity of the tricuspid annulus (S’) [18,19].

#### 2.2.3. Evaluation of PE Extent by CT-PA

PE was classified as central when filling defects involved the pulmonary artery trunk, the main pulmonary arteries, or the lobar branches, and as peripheral when limited to segmental or subsegmental branches.

### 2.3. Statistical Analysis

Sample size was calculated assuming an OSA prevalence of 40% in the control group, an odds ratio of 2.5, a significance level of 5%, and a power of 80%. All patients who met the selection criteria during the recruitment period were included, with an approximate ratio of two controls per case. The final sample analyzed consisted of 128 patients with PE and 252 controls.

Qualitative variables were expressed as absolute frequencies and percentages, and quantitative variables as mean and standard deviation. Comparisons between groups were performed using Pearson’s chi-square or Fisher’s exact test for qualitative variables and the Mann–Whitney U test for quantitative variables.

To evaluate the association of the AHI with the presence of PE and with the severity of the episode, logistic regression models were used, initially crude and subsequently adjusted for the variables considered of interest. For the development of chronic DVT and VTE recurrence, only univariate logistic regression analyses were performed due to the limited number of events. The overall contribution of the models was evaluated using the likelihood ratio test and their discriminative capacity using the area under the ROC curve with its 95% confidence interval.

Model I included age, sex, and BMI, selected a priori because of their established association with OSA and their potential role as confounding factors. Model II additionally included selected VTE-related risk factors and relevant comorbidities available in the dataset, based on their clinical relevance to the association under study. The number of covariates included in Model II was limited according to the available sample size and number of events to reduce the risk of overfitting. Neck circumference was not systematically measured and therefore could not be included in the models.

Age, sex, and BMI were included a priori because of their established clinical relevance as potential confounders of the association between OSA and PE. Additional VTE-related risk factors and comorbidities were considered in the second model based on their clinical relevance and observed distribution between groups.

Values of *p* < 0.05 were considered statistically significant. Analyses were performed with IBM SPSS Statistics, version 29, and R, version 4.4.2.

## 3. Results

### 3.1. Description of the Study Population

**Figure 1:**
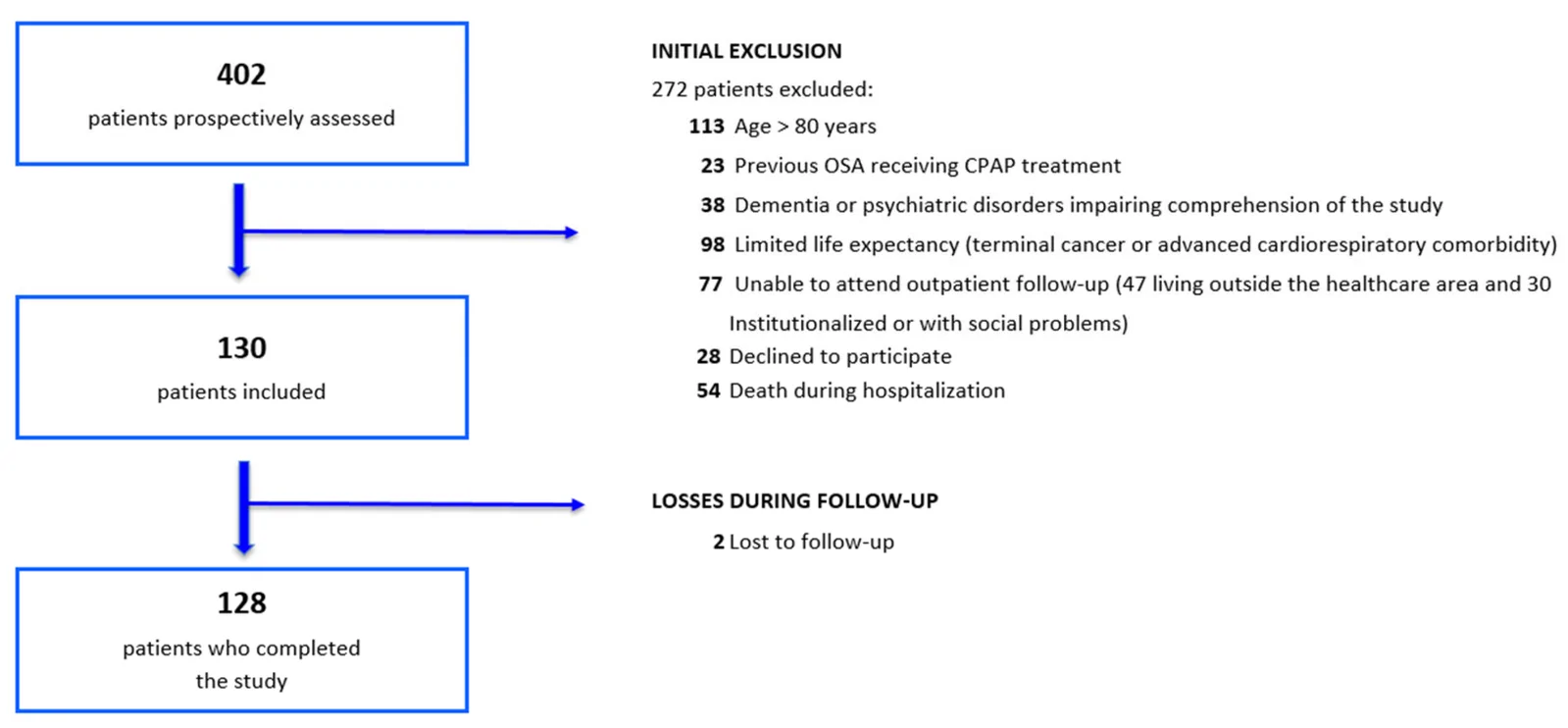
Selection and inclusion flow diagram of the patients in the case group.

A total of 402 patients admitted for acute PE were evaluated prospectively. After applying the selection criteria, 130 patients were included; 2 were lost during follow-up, so 128 completed the study. Figure 1 shows the selection and inclusion flow diagram of the patients in the case group.

**Figure 2:**
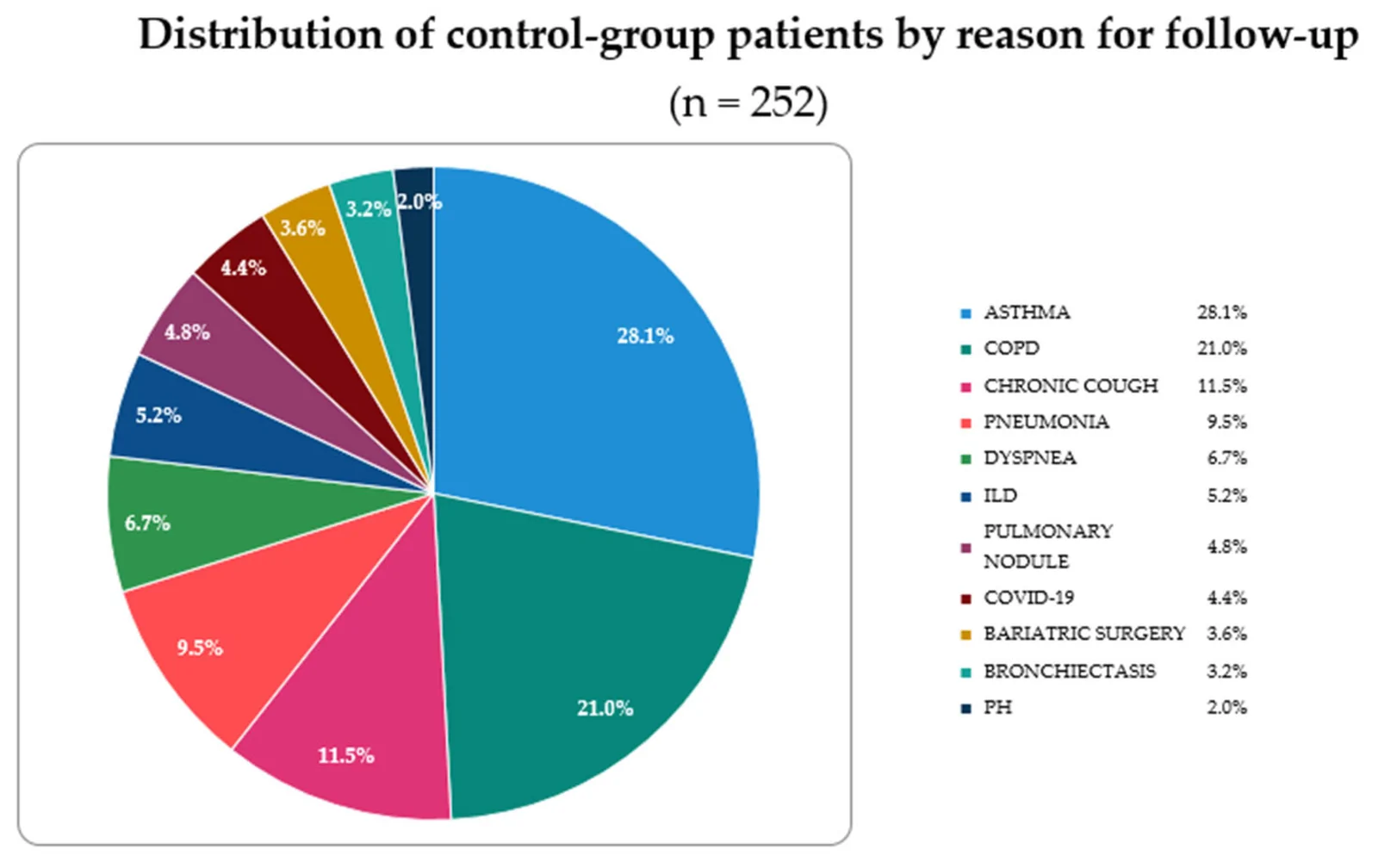
Distribution of the patients in the control group according to the reason for follow-up in pulmonology clinics. COPD, chronic obstructive pulmonary disease; ILD, interstitial lung disease; PH, pulmonary hypertension.

In the control group, 260 outpatients followed in pulmonology clinics for respiratory diseases other than PE were initially included; 8 refused respiratory polygraphy, so the final sample consisted of 252 controls. Figure 2 shows the distribution of the patients in the control group according to the reason for follow-up in pulmonology clinics.

### 3.2. Clinical Characteristics of PE Patients on Admission, According to the Presence of OSA

Among the 128 patients with acute PE, the prevalence of OSA was 55.5% (71 patients) and 28.9% (37 patients) had severe OSA.

Baseline characteristics, comorbidities, VTE risk factors, and clinical parameters on admission are summarized in Table 1 and Table 2. Patients with OSA were significantly older than those without OSA (67 ± 12 vs. 57 ± 15 years; *p* < 0.001) and had a higher prevalence of obesity (71.8% vs. 52.6%; *p* = 0.025), hypertension (60.6% vs. 36.8%; *p* = 0.008), resistant hypertension (19.7% vs. 7.0%; *p* = 0.040), and cardiovascular comorbidity (28.2% vs. 12.3%; *p* = 0.029).

No significant differences were observed in sex distribution, smoking status, or use of sedative medication.

Regarding VTE risk factors, the proportion of unprovoked PE was similar in both groups. Prolonged immobilization and active cancer were more frequent in patients with OSA (12.7% vs. 1.8%; *p* = 0.042, and 12.7% vs. 0%; *p* = 0.004, respectively), while the use of oral contraceptives was more frequent in patients without OSA (12.3% vs. 1.4%; *p* = 0.022).

Regarding the initial severity of PE, syncope and acute hypoxemic respiratory failure (defined by baseline SpO_2_ < 90%) were significantly more frequent in patients with OSA (16.9% vs. 5.3%; *p* = 0.042 and 39.4% vs. 15.8%; *p* = 0.003, respectively). Likewise, the proportion of patients with PESI ≥ III was also higher in this group (69.0% vs. 43.9%; *p* = 0.004). According to ESC prognostic stratification, a higher proportion of patients without OSA was classified as low risk (49.1% vs. 25.4%; *p* = 0.035). No significant differences were observed in thrombotic extent on CT-PA, presence of DVT, cardiac biomarkers, RV dysfunction on TTE, or administration of fibrinolytic treatment.

### 3.3. Clinical Evolution During Follow-Up According to the Presence of OSA

The clinical evolution of the patients during follow-up is summarized in Table 3. No significant differences were observed between patients with and without OSA in the development of chronic DVT, chronic PE, or chronic thromboembolic pulmonary hypertension, nor in the functional parameters evaluated using the diffusing capacity of the lungs for carbon monoxide (DLCO) and the 6-min walk test (6MWT).

Withdrawal of anticoagulant treatment was less frequent in patients with OSA than in those without OSA (35.2% vs. 52.6%; *p* = 0.048). However, among patients in whom anticoagulation was discontinued, VTE recurrence during the year after its withdrawal was significantly more frequent in the OSA group (36.0% vs. 13.3%; *p* = 0.049).

No significant differences were observed in the diagnosis of neoplasms or in mortality during the year after the PE episode.

### 3.4. Association of AHI with the Presence of PE

Table 4 shows the results of the logistic regression analysis performed on the 380 study participants, including 128 patients with PE and 252 controls without PE. Each 10 events/h increase in AHI was associated with the presence of PE in the crude analysis (OR = 1.259; 95% CI: 1.107–1.433; *p* < 0.001). This association persisted after adjusting for age, sex, and BMI (OR = 1.162; 95% CI: 1.005–1.343; *p* = 0.042) and was maintained in a broader model that included several recognized VTE risk factors (OR = 1.205; 95% CI: 1.019–1.426; *p* = 0.029).

### 3.5. Association of AHI with PE Severity

Table 5 shows the results of the logistic regression analysis performed in the 128 PE patients, of whom 34 had an intermediate–high or high-risk PE. Each 10 events/h increase in AHI showed a non-significant association with this outcome in the crude analysis (OR = 1.205; 95% CI: 0.999–1.466; *p* = 0.063). After adjusting for age, sex, and BMI, the association remained non-significant (OR = 1.173; 95% CI: 0.939–1.465; *p* = 0.159).

### 3.6. Association of AHI with the Development of Chronic DVT

Table 6 shows the results of the univariate logistic regression analyses performed in the 128 PE patients, of whom 24 developed chronic DVT during follow-up. AHI, expressed per each 10 events/h increase, was not significantly associated with this outcome (OR = 1.093; 95% CI: 0.877–1.363; *p* = 0.429). No statistically significant associations were observed with the rest of the variables analyzed. Due to the limited number of events, no multivariate model was fitted.

### 3.7. Association of AHI with Recurrence of Venous Thromboembolic Disease

Table 7 presents the results of the univariate logistic regression analyses performed in the 55 patients in whom anticoagulant treatment was withdrawn, of whom 13 had a VTE recurrence during the following year. Among the 55 patients in whom anticoagulation was discontinued, 20 (36.4%) had a low-risk unprovoked PE, whereas the remaining cases were associated with identifiable risk factors: 7 (12.7%) with oral contraceptive use, 11 (20.0%) with recent surgery or trauma, 4 (7.3%) with recent air travel, 10 (18.2%) with recent COVID-19 infection, and 3 (5.5%) with thrombophilia. The reasons for anticoagulation withdrawal were based on radiological findings of PE, absence of VTE, and absence of pulmonary hypertension.

The increase in AHI, expressed per each 10 events/h, showed a non-significant association with recurrence (OR = 1.393; 95% CI: 0.988–1.964; *p* = 0.059). No statistically significant associations were observed with the rest of the variables analyzed. Due to the limited number of events, no multivariate model was fitted.

### 3.8. Comparison of Baseline Characteristics, OSA Prevalence and Related Symptoms Between Cases and Controls

Baseline characteristics and comorbidities of the 128 patients with PE and the 252 controls are summarized in Table 8. PE patients had a higher prevalence of obesity (63.3% vs. 46.0%; *p* = 0.001) and sedentary lifestyle (31.3% vs. 17.5%; *p* = 0.002). In contrast, history of smoking (68.3% vs. 55.5%; *p* = 0.014) and cumulative tobacco consumption (22.13 vs. 15.33 pack-years; *p* = 0.003) were more frequent in the controls. No significant differences were observed between groups in age or sex distribution. Regarding comorbidities, COPD was more frequent in controls (23.8% vs. 14.1%; *p* = 0.026), while ENT (otorhinolaryngological) pathology associated with OSA was more frequent in PE patients (34.1% vs. 21.9%; *p* = 0.014). No differences were observed in the remaining comorbidities or in the use of sedative medication.

Regarding OSA-related symptoms, PE patients presented more frequently witnessed apneas, excessive daytime sleepiness, tiredness, and memory or concentration impairment, as shown in Table 9.

The results of the respiratory polygraphy are shown in Table 9. PE patients had a higher mean AHI than the controls (21.21 ± 19.29 vs. 14.81 ± 14.26 events/h; *p* = 0.007) and a higher ODI (19.86 ± 18.77 vs. 13.30 ± 13.79; *p* = 0.003). However, TC90 was lower in cases than in controls (11.75 ± 17.12 vs. 20.58 ± 27.74; *p* = 0.029). The prevalence of OSA was higher in PE patients (55.5% vs. 42.1%; *p* = 0.013). When analyzing the distribution according to OSA severity, controls presented mild OSA more frequently (32.9% vs. 22.7%; *p* = 0.038), while severe OSA was more frequent in PE patients (28.9% vs. 13.9%; *p* = 0.001).

## 4. Discussion

The main finding of this study is the high prevalence of previously undiagnosed obstructive sleep apnea (OSA) among patients with acute pulmonary embolism (PE). More than half of the patients with PE had OSA (55.5%), and almost one-third had severe disease (28.9%). Importantly, both the prevalence and severity of OSA were higher than those observed in our respiratory control group. These findings reinforce the potential clinical relevance of sleep-disordered breathing in patients with PE and support consideration of targeted OSA assessment during follow-up.

Previous studies have reported an association between OSA and venous thromboembolism (VTE), although the magnitude of this relationship remains uncertain. A recent systematic review and meta-analysis highlighted substantial heterogeneity in reported OSA prevalence among patients with VTE, partly attributable to differences in study populations and, importantly, in the timing of sleep assessment relative to the thromboembolic event [8]. Our study attempted to address this issue by performing respiratory polygraphy at least three months after PE. This timing is relevant because sleep-disordered breathing may be transiently influenced by the acute thromboembolic event. Previous studies have shown that the frequency and severity of obstructive respiratory events may increase during the acute phase of PE and subsequently decrease during clinical stabilization, particularly in patients with right ventricular dysfunction [15,16]. Therefore, studies performed shortly after PE may overestimate the underlying prevalence or severity of OSA.

The clinical characteristics of patients with OSA were broadly consistent with the phenotype typically associated with this disorder. Patients with OSA were older and more frequently obese and had a higher prevalence of hypertension, resistant hypertension, and cardiovascular comorbidity. Active cancer was also more frequent among patients with OSA, representing an additional potential confounder because malignancy is an established risk factor for VTE. These differences emphasize the importance of accounting for shared risk factors when assessing the relationship between OSA and PE.

Despite these potential confounders, the association between AHI and PE remained statistically significant after adjustment for age, sex, BMI, and multiple established VTE risk factors. Each 10-events/h increase in AHI was associated with 20.5% higher odds of PE in the fully adjusted model (OR 1.205, 95% CI 1.019–1.426; *p* = 0.029). This finding is consistent with previous epidemiological studies suggesting an association between OSA and incident VTE [7,9,10] and with the broader literature describing an association between OSA and thromboembolic risk [5,6]. Several mechanisms could plausibly underlie this relationship, including intermittent hypoxia, sympathetic activation, oxidative stress, systemic inflammation, endothelial dysfunction, and a prothrombotic state [5,6]. Nevertheless, the observational design of our study precludes establishing a causal relationship between OSA severity and PE.

An additional finding was the greater clinical severity observed among PE patients with OSA. Syncope, baseline hypoxemia, and a PESI score ≥ III were more frequent in patients with OSA, while patients without OSA were more frequently classified as low risk according to ESC prognostic stratification. However, this apparent relationship was not supported by the specific analysis of AHI and PE severity. Increasing AHI was not independently associated with intermediate–high or high-risk PE after adjustment for age, sex, and BMI. Moreover, no significant differences were observed in thrombotic extent, deep vein thrombosis (DVT), cardiac biomarkers, right ventricular dysfunction, or fibrinolytic treatment. These findings suggest that the greater clinical severity observed in patients with OSA may be partly explained by differences in age, obesity, and comorbidity rather than by an independent effect of OSA itself. This interpretation is consistent with the heterogeneous results reported in previous studies evaluating the relationship between OSA and PE severity [8].

Our findings regarding chronic thromboembolic evolution were similarly negative. Neither the presence of OSA nor increasing AHI was significantly associated with chronic DVT, and no differences were observed in chronic PE, chronic thromboembolic pulmonary hypertension, or subsequent functional impairment assessed by DLCO and the 6-min walk test. The lack of association between AHI and chronic DVT (OR 1.093 per 10-events/h increase; 95% CI 0.877–1.363; *p* = 0.429) does not support a major role for OSA severity in thromboembolic persistence. However, these results should be interpreted cautiously because the number of chronic events was limited and multivariable analysis was not feasible.

In contrast, a clinically relevant signal emerged regarding VTE recurrence. Anticoagulation was discontinued less frequently in patients with OSA than in those without OSA. Among patients in whom anticoagulation was withdrawn, however, recurrence during the subsequent year was significantly more frequent in those with OSA (36.0% vs. 13.3%; *p* = 0.049). Furthermore, increasing AHI showed a potentially relevant association with recurrence (OR 1.393 per 10-events/h increase), although statistical significance was not reached (*p* = 0.059). These findings are intriguing in view of the established risk of recurrent VTE after anticoagulation discontinuation [3] and the proposed prothrombotic effects of OSA [5,6]. Nevertheless, only 55 patients discontinued anticoagulation and 13 recurrences occurred, precluding multivariable analysis. Accordingly, OSA and AHI cannot currently be considered independent predictors of recurrent VTE, and these observations should be regarded as exploratory and hypothesis-generating and interpreted with caution.

The comparison with respiratory controls provides an additional strength. This design provides complementary information to previous pooled evidence by allowing a direct comparison with a clinically characterized respiratory control group assessed using the same diagnostic approach and by evaluating the independent association between AHI and PE after adjustment for relevant confounders. OSA prevalence was significantly higher among PE patients than controls (55.5% vs. 42.1%), with a particularly marked difference in severe OSA (28.9% vs. 13.9%). This comparison is clinically relevant because respiratory disorders such as COPD, asthma, and bronchiectasis are themselves associated with an increased prevalence of OSA [20,21,22]. The groups were similar in age and sex, although PE patients were more frequently obese, whereas controls had greater tobacco exposure and a higher prevalence of COPD. These differences may have contributed to the observed findings and highlight the possibility of residual confounding.

Interestingly, PE patients had higher AHI and oxygen desaturation index values, but lower TC90 than the controls. This apparent discrepancy contrasts with studies suggesting that nocturnal hypoxemia, particularly TC90, may be more closely related to VTE risk than AHI [9,10]. The higher prevalence of COPD among controls may partly explain their greater nocturnal hypoxemia independently of obstructive events. PE patients also reported more witnessed apneas, excessive daytime sleepiness, pathological Epworth scores, daytime tiredness, and cognitive complaints, suggesting that the increased OSA burden was clinically meaningful rather than solely a polygraphic finding.

This study has several strengths, including its prospective design, consecutive recruitment of PE patients, standardized assessment, delayed sleep evaluation after the acute phase, and adjustment for multiple potential confounders. The inclusion of a respiratory control group also provides a clinically relevant comparison that is uncommon in previous studies. Nevertheless, several limitations should be acknowledged. The single-center design and exclusion of patients older than 80 years or with limited life expectancy may restrict generalizability. Controls were recruited from respiratory clinics and therefore do not represent the general population. Neck circumference was not systematically measured and therefore could not be included in the multivariable analyses. Although BMI was included as an objective measure of adiposity and an important OSA-related confounder, BMI may not fully capture obesity-related factors, including body fat distribution and upper-airway anatomy; therefore, the independent effect of obesity-related factors may not be fully captured by BMI adjustment and residual confounding cannot be completely excluded. Residual confounding cannot be excluded, particularly given differences in obesity, COPD, and cancer. Importantly, OSA was assessed at the 3–6-month follow-up visit but was not systematically reassessed after documented resolution of PE; therefore, persistence of OSA independently of residual thromboembolic disease cannot be definitively established. Moreover, because OSA was assessed after the acute PE episode, the temporal direction of the observed association cannot be established, and the present observational design does not allow causal inference. Finally, the limited number of follow-up events prevented multivariable analyses of chronic DVT and VTE recurrence, and OSA was diagnosed using home respiratory polygraphy rather than polysomnography, although the same protocol was applied to both groups and recordings were manually reviewed by blinded pulmonologists.

## 5. Conclusions

In conclusion, our results show a high prevalence of undiagnosed OSA among patients who have experienced an acute PE, with a high proportion of severe OSA, higher than that observed in our control group and that described in previous general-population studies. Increased AHI was independently associated with the presence of PE after adjusting for multiple confounding factors, which supports the hypothesis of a possible relationship between a higher AHI and thromboembolic disease. In contrast, no independent association of AHI was demonstrated with the initial severity of PE or with the development of chronic DVT. The higher frequency of recurrences after withdrawal of anticoagulation observed among OSA patients and the trend toward greater recurrence with increasing AHI constitute findings that require confirmation in larger studies. Taken together, these results support a clinically relevant association between OSA and PE and reinforce the importance of considering a targeted search for OSA during follow-up, especially in patients with suggestive symptoms or clinical characteristics.

## Figures and Tables

**Table 1:** Baseline characteristics, comorbidities and venous thromboembolic disease risk factors according to the presence of OSA in PE patients. Data are expressed as n (%) or mean ± standard deviation. OSA: obstructive sleep apnea; BMI: body mass index; VTE: venous thromboembolic disease; PE: pulmonary thromboembolism. * Fisher’s exact test.

	No OSA (n = 57)	OSA (n = 71)	*p*
**Baseline characteristics**			
Age (years)	57.18 ± 15.45	66.62 ± 11.93	<0.001
Male sex	31 (54.4%)	44 (62.0%)	0.387
Obesity (BMI ≥ 30 kg/m^2^)	30 (52.6%)	51 (71.8%)	0.025
History of smoking	33 (57.9%)	38 (53.5%)	0.621
Active smoker	11 (19.3%)	9 (12.7%)	0.305
Sedentary lifestyle	13 (22.8%)	27 (38.0%)	0.065
Use of sedative medication	23 (40.4%)	27 (38.0%)	0.789
**Comorbidities**			
Hypertension	21 (36.8%)	43 (60.6%)	0.008
Resistant hypertension	4 (7.0%)	14 (19.7%)	0.040
Diabetes mellitus	5 (8.8%)	13 (18.3%)	0.123
Cardiovascular comorbidity	7 (12.3%)	20 (28.2%)	0.029
Chronic venous insufficiency	24 (42.1%)	36 (50.7%)	0.333
Chronic respiratory disease	18 (31.6%)	16 (22.5%)	0.250
**Thromboembolic risk factors**			
Recent surgery	3 (5.3%)	7 (9.9%)	0.510 *
Recent trauma	1 (1.8%)	3 (4.2%)	0.628 *
Prolonged non-surgical immobilization	1 (1.8%)	9 (12.7%)	0.042 *
Air travel	5 (8.8%)	2 (2.8%)	0.241 *
Active cancer	0 (0.0%)	9 (12.7%)	0.004 *
Prior VTE	8 (14.0%)	7 (9.9%)	0.465
Oral contraceptives	7 (12.3%)	1 (1.4%)	0.022 *
Recent COVID-19	13 (22.8%)	10 (14.1%)	0.201
Thrombophilia	7 (12.3%)	7 (9.9%)	0.663
Unprovoked PE	23 (40.4%)	31 (43.7%)	0.706

**Table 2:** Clinical characteristics, thrombotic extent, biomarkers, echocardiographic findings and prognostic stratification of PE according to the presence of OSA. Data are expressed as n (%) or mean ± standard deviation. OSA: obstructive sleep apnea; SpO_2_: oxygen saturation; SBP: systolic blood pressure; HR: heart rate; PESI: Pulmonary Embolism Severity Index; PE: pulmonary thromboembolism; DVT: deep vein thrombosis; NT-proBNP: N-terminal pro–B-type natriuretic peptide; RV: right ventricle; SPAP: systolic pulmonary artery pressure; TAPSE: tricuspid annular plane systolic excursion. * Fisher’s exact test.

	No OSA (n = 57)	OSA (n = 71)	*p*
**Clinical presentation and severity**			
Syncope	3 (5.3%)	12 (16.9%)	0.042
Baseline SpO_2_ < 90%	9 (15.8%)	28 (39.4%)	0.003
SBP < 100 mmHg	4 (7.0%)	3 (4.2%)	0.699 *
HR > 110 bpm	15 (26.3%)	24 (33.8%)	0.360
PESI:			
PESI I	18 (31.6%)	4 (5.6%)	
PESI II	14 (24.6%)	18 (25.4%)	
PESI III	15 (26.3%)	24 (33.8%)	0.002
PESI IV	5 (8.8%)	14 (19.7%)	
PESI V	5 (8.8%)	11 (15.5%)	
PESI ≥ III	25 (43.9%)	49 (69.0%)	0.004
**Thrombotic extent**			
Central PE	31 (54.4%)	50 (70.4%)	0.061
DVT	34 (59.6%)	38 (53.5%)	0.487
Proximal DVT	19 (33.3%)	27 (38.0%)	0.582
**Biomarkers and RV involvement**			
Elevated cardiac biomarkers (high-sensitivity troponin T and NT-proBNP)	14 (24.6%)	27 (38.0%)	0.105
Right ventricular dysfunction	17 (29.8%)	29 (40.8%)	0.197
PHT (SPAP ≥ 40 mmHg)	16 (28.1%)	25 (35.2%)	0.389
Moderate–severe PHT (SPAP ≥ 50 mmHg)	12 (21.1%)	15 (21.1%)	0.992
Decreased TAPSE (<16 mm)	6 (10.5%)	11 (14.1%)	0.411
RV dilation	10 (17.5%)	18 (25.4%)	0.288
RV hypokinesia	6 (10.5%)	10 (14.1%)	0.545
Other signs of RV dysfunction	9 (15.8%)	14 (19.7%)	0.565
**Prognostic stratification**			
Risk:			
Low risk	28 (49.1%)	18 (25.4%)	0.035 *
Intermediate–low risk	17 (29.8%)	31 (43.7%)	
Intermediate–high risk	10 (17.5%)	20 (28.2%)	
High risk	2 (3.5%)	2 (2.8%)	
Fibrinolysis	1 (1.8%)	2 (2.8%)	1.000 *

**Table 3:** Clinical evolution during follow-up according to the presence of OSA in PE patients. Data are expressed as n (%). OSA: obstructive sleep apnea; DVT: deep vein thrombosis; PE: pulmonary thromboembolism; CTEPH: chronic thromboembolic pulmonary hypertension; DLCO: diffusing capacity of the lungs for carbon monoxide; 6MWT: 6-min walk test; VTE: venous thromboembolic disease. † Calculated only among patients in whom anticoagulant treatment was withdrawn. * Fisher’s exact test.

	No OSA (n = 57)	OSA (n = 71)	*p*
Chronic DVT	10 (17.5%)	14 (19.7%)	0.754
Chronic PE	3 (5.3%)	3 (4.2%)	1.000 *
Moderate–severe chronic thromboembolic pulmonary hypertension	0 (0%)	1 (1.4%)	1.000 *
Decreased DLCO	7 (14%)	12 (22.2%)	0.278
Normal 6-min walk distance	41 (87.2%)	40 (80.0%)	0.337
Desaturation during 6MWT	1 (2.1%)	1 (2.0%)	1.000 *
Withdrawal of anticoagulation	30 (52.6%)	25 (35.2%)	0.048
VTE recurrence after anticoagulation withdrawal †	4 (13.3%)	9 (36%)	0.049
Diagnosis of neoplasm in the following year	4 (7%)	5 (7%)	1.000 *
Death in the following year	3 (5.3%)	7 (9.9%)	0.528 *

**Table 4:** Association between the apnea–hypopnea index and the presence of pulmonary thromboembolism: logistic regression models. AHI: apnea–hypopnea index; CI: confidence interval. N = 380; PE = 128; controls = 252. * Per each 10 events/h increase in AHI. Model I: adjusted for age, sex and BMI. Model II: adjusted for sex, age, BMI, smoking, sedentary lifestyle, cardiovascular comorbidity, chronic venous insufficiency, surgery, trauma, non-surgical immobilization, active cancer, oral contraceptives and prior COVID-19.

	Crude Model − OR (95% CI)	Crude Model *p*-Value	Model I − OR (95% CI)	Model I *p*-Value	Model II − OR (95% CI)	Model II *p*-Value
AHI *	1.259 (1.107–1.433)	<0.001	1.162 (1.005–1.343)	0.042	1.205 (1.019–1.426)	0.029

**Table 5:** Association between the apnea–hypopnea index and the presence of intermediate–high or high-risk PE: logistic regression models. AHI: apnea–hypopnea index; OR: odds ratio; CI: confidence interval. N = 128; intermediate–high or high-risk PE = 34. * Per each 10 events/h increase in AHI. Model I: adjusted for age, sex and BMI.

	**Crude Model − OR (95% CI)**	***p*** **-Value**
AHI *	1.205 (0.999–1.466)	0.063
	**Model I − OR (95% CI)**	***p*** **-Value**
AHI *	1.173 (0.939–1.465)	0.159

**Table 6:** Factors associated with the development of chronic DVT in PE patients: univariate logistic regression analysis. N = 128; chronic DVT = 24. * Per each 10 events/h increase in AHI. NE: not estimable due to the small number of observations or events in this category; OR: odds ratio; CI: confidence interval; AHI: apnea–hypopnea index; VTE: venous thromboembolic disease; PE: pulmonary thromboembolism.

Crude Model	OR	95% CI	*p*-Value
AHI *	1.093	(0.877–1.363)	0.429
Age > 65 years	1.400	(0.570–3.436)	0.463
Male sex	1.926	(0.736–5.038)	0.181
Obesity	0.773	(0.313–1.912)	0.577
Sedentary lifestyle	1.125	(0.437–2.895)	0.807
Chronic venous insufficiency	2.185	(0.877–5.445)	0.093
Active cancer	NE	NE	NE
Thrombophilia	1.880	(0.535–6.601)	0.325
Prior VTE	1.691	(0.488–5.856)	0.407
Unprovoked PE	1.816	(0.743–4.440)	0.191

**Table 7:** Factors associated with VTE recurrence after withdrawal of anticoagulation: univariate logistic regression analysis. n = 55 patients in whom anticoagulation were withdrawn; VTE recurrence = 13. * Per each 10 events/h increase in AHI. NE: not estimable due to the small number of observations or events in this category; OR: odds ratio; CI: confidence interval; AHI: apnea–hypopnea index; VTE: venous thromboembolic disease; PE: pulmonary thromboembolism.

Crude Model	OR	95% CI	*p*-Value
AHI *	1.393	(0.988–1.964)	0.059
Age > 65 years	3.200	(0.882–11.60)	0.077
Male sex	0.943	(0.271–3.282)	0.926
Obesity	1.250	(0.329–4.756)	0.743
Sedentary lifestyle	1.100	(0.249–4.861)	0.900
Chronic venous insufficiency	2.416	(0.665–8.769)	0.180
Active cancer	1.667	(0.139–20.01)	0.687
Thrombophilia	1.667	(0.139–20.01)	0.687
Prior VTE	NE	NE	NE
Unprovoked PE	1.563	(0.425–5.748)	0.502

**Table 8:** Comparison of baseline characteristics and comorbidities between PE patients and the control group. Data are expressed as n (%) or mean ± standard deviation. PE: pulmonary thromboembolism; BMI: body mass index; COPD: chronic obstructive pulmonary disease; ENT: otorhinolaryngological; OSA: obstructive sleep apnea.

	Controls (n = 252)	PE (n = 128)	*p*
**Baseline characteristics**			
Age (years)	59.96 ± 14.19	62.41 ± 14.35	0.070
Male sex	121 (48.0%)	75 (58.6%)	0.051
Obesity (BMI ≥ 30 kg/m^2^)	116 (46.0%)	81 (63.3%)	0.001
History of smoking	172 (68.3%)	71 (55.5%)	0.014
Active smoker	60 (23.8%)	20 (15.6%)	0.064
Pack-year index	22.13 ± 23.13	15.33 ± 20.40	0.003
Sedentary lifestyle	44 (17.5%)	40 (31.3%)	0.002
Use of sedative medication	86 (34.1%)	50 (39.1%)	0.343
**Comorbidities**			
Hypertension	119 (47.2%)	64 (50%)	0.609
Resistant hypertension	29 (11.5%)	18 (14.1%)	0.475
Diabetes mellitus	42 (16.7%)	18 (14.1%)	0.511
Dyslipidemia	111 (44.0%)	54 (42.2%)	0.730
Cardiovascular comorbidity	44 (17.5%)	27 (21.1%)	0.390
COPD	60 (23.8%)	18 (14.1%)	0.026
ENT pathology associated with OSA	55 (21.9%)	44 (34.1%)	0.014

**Table 9:** OSA-related symptoms, respiratory polygraphy parameters and prevalence of OSA in PE patients and controls. PE: pulmonary thromboembolism; OSA: obstructive sleep apnea; ESS: Epworth Sleepiness Scale; AHI: apnea–hypopnea index; ODI: Oxygen Desaturation Index; TC90: mean percentage of sleep time with arterial oxygen saturation measured by pulse-oximetry below 90%; SpO_2_: saturation of peripheral oxygen.

	Controls (n = 252)	PE (n = 128)	*p*
**OSA-related symptoms**			
Snoring	197 (78.2%)	106 (82.8%)	0.288
Witnessed apneas	51 (20.2%)	38 (29.7%)	0.040
Excessive daytime sleepiness	46 (18.3%)	40 (31.3%)	0.004
ESS > 10	44 (17.5%)	37 (28.9%)	0.010
Daytime tiredness	116 (46.0%)	73 (57.0%)	0.043
Frequent morning headache	35 (13.9%)	22 (17.2%)	0.395
Non-restorative sleep	124 (49.2%)	67 (52.3%)	0.563
Nocturnal choking crises	34 (13.5%)	12 (9.4%)	0.245
Nocturia	96 (38.1%)	61 (47.7%)	0.074
Memory/concentration impairment	67 (26.6%)	49 (38.3%)	0.019
Irritability/mood changes	65 (25.8%)	41 (32.0%)	0.200
**Respiratory polygraphy parameters and OSA prevalence**			
AHI	14.81 ± 14.26	21.21 ± 19.29	0.007
ODI	13.30 ± 13.79	19.86 ± 18.77	0.003
TC90	20.58 ± 27.74	11.75 ± 17.12	0.029
Mean SpO_2_	91.57 ± 2.86	92.52 ± 2.14	0.001
Diagnosis of OSA	106 (42.1%)	71 (55.5%)	0.013
AHI < 5	76 (30.2%)	33 (25.8%)	0.373
AHI 5–14.9	83 (32.9%)	29 (22.7%)	0.038
AHI d15–29.9	58 (23%)	29 (22.7%)	0.937
AHI ≥ 30	35 (13.9%)	37 (28.9%)	0.001

## Data Availability

The original contributions presented in this study are included in the article. Further inquiries can be directed to the corresponding author.

## Abbreviations

The following abbreviations are used in this manuscript:

AHI	Apnea–Hypopnea Index
BMI	Body Mass Index
COPD	Chronic Obstructive Pulmonary Disease
CPAP	Continuous Positive Airway Pressure
CTPA	Computed Tomography Pulmonary Angiography
DLCO	Diffusing Capacity of the Lung for Carbon Monoxide
DVT	Deep Vein Thrombosis
ESC	European Society of Cardiology
ESS	Epworth Sleepiness Scale
HR	Heart Rate
ODI	Oxygen Desaturation Index
OSA	Obstructive Sleep Apnea
PE	Pulmonary Embolism
PESI	Pulmonary Embolism Severity Index
RV	Right Ventricle
SBP	Systolic Blood Pressure
SpO_2_	Peripheral Oxygen Saturation
SPAP	Systolic Pulmonary Artery Pressure
TC90	Percentage of Recording Time with Oxygen Saturation <90%
TAPSE	Tricuspid Annular Plane Systolic Excursion
TTE	Transthoracic Echocardiography
VTE	Venous Thromboembolism
